# Novel environments induce variability in fitness‐related traits

**DOI:** 10.1002/ece3.10165

**Published:** 2023-06-06

**Authors:** Arielle W. Balph, Amy C. Krist

**Affiliations:** ^1^ Department of Zoology and Physiology University of Wyoming Laramie Wyoming USA; ^2^ Program in Ecology and Evolution University of Wyoming Laramie Wyoming USA

**Keywords:** novel environments, phenotypic plasticity, Physidae, variation

## Abstract

Environmental change from anthropogenic activities threatens individual organisms, the persistence of populations, and entire species. Rapid environmental change puts organisms in a double bind, they are forced to contend with novel environmental conditions but with little time to respond. Phenotypic plasticity can act quickly to promote establishment and persistence of individuals and populations in novel or altered environments. In typical environmental conditions, fitness‐related traits can be buffered, reducing phenotypic variation in expression of traits, and allowing underlying genetic variation to accumulate without selection. In stressful conditions, buffering mechanisms can break down, exposing underlying phenotypic variation, and permitting the expression of phenotypes that may allow populations to persist in the face of altered or otherwise novel environments. Using reciprocal transplant experiments of freshwater snails, we demonstrate that novel conditions induce higher variability in growth rates and, to a lesser degree, morphology (area of the shell opening) relative to natal conditions. Our findings suggest a potentially important role of phenotypic plasticity in population persistence as organisms face a rapidly changing, human‐altered world.

## Graphical abstract

Snails expressed much greater variability in specific growth rates in novel, transplanted sites relative to their natal site (a; *p* < .001). Populations expressed moderately higher variability in aperture area in novel sites relative to their natal site (b; *p* ≤ .020) and no difference in variability in aperture shape (ratio of aperture width to aperture length) between natal and novel sites (c; *p* > .5).

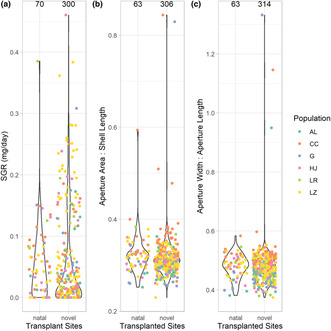

## 1 INTRODUCTION

Globally, anthropogenic activities are severely altering terrestrial and aquatic environments. Freshwater ecosystems face alterations to water levels and flow regimes via climate change, contamination from fertilizers and other pollutants, habitat destruction, and the introduction of non‐native species (Cordellier et al., 2012; Dextrase & Mandrak, 2006). Disturbed aquatic habitats also propagate the spread of invasive species and promote range expansions of invasive and native species (Parmesan et al., 2003; Rahel et al., 2008; Ruppert et al., 2017). When environmental change is rapid, organisms have little time to respond.

Phenotypic plasticity can promote establishment and persistence of individuals and populations in novel or altered environments. For organisms facing rapid anthropogenic change, plasticity can act quickly before directional selection can alter allele frequencies (Crispo, 2007; Ghalambor et al., 2007; Kilvitis et al., 2017; Lande, 2009). Behavioral plasticity can occur almost immediately (Ghalambor et al., 2010; West‐Eberhard, 1989). For example, canary chicks can increase their begging behavior in response to short‐term hunger or lower body condition (Ghalambor et al., 2010). While life history and morphological plasticity take longer to occur, they still occur within an individual's lifetime (Ghalambor et al., 2010; West‐Eberhard, 1989). For example, pumpkinseed fish‐fed gastropods develop stronger jaw muscles than pumpkinseed fish‐fed soft‐bodied prey (Mittelbach et al., 1999), and guppies reared on low food quantities mature at a later age than guppies reared on higher quantities of food (Auer, 2010).

Natural selection can either reduce or enhance phenotypic plasticity. Reduced plasticity in fitness‐related traits can occur in natal conditions through genetic assimilation or canalization (Crispo, 2007; Waddington, 1942). Alternatively, variation in fitness‐related traits can increase via accumulation of “cryptic genetic variation” (Ghalambor et al., 2007; Rutherford, 2000): expression of phenotypic variants is buffered in natal conditions allowing mutations to accumulate without natural selection acting on unexpressed variants (Buckley et al., 2010; Rutherford, 2000; Waddington, 1942). When organisms are released from natal regulatory cues or encounter stressful conditions, buffering mechanisms can break down and expose the underlying phenotypic variation in these traits (Paaby & Rockman, 2014; Rutherford, 2000; Schlichting, 2008). Examples include variability in sperm performance in brown trout induced by stressful pH (Purchase & Moreau, 2012) and temperature shocks increasing variation in wing spot variation of the pale grass blue butterfly (Buckley et al., 2010) and sperm storage organs of the yellow dung fly (Berger et al., 2011). Hidden variation revealed by novel conditions can increase variance in traits leading to negative, neutral, or positive effects on individual fitness in the new environment. Thus, it could permit the expression of some phenotypes that allow populations to persist in the face of altered or otherwise novel environments.

To examine whether novel environments increased trait variation in natural populations of freshwater snails, we contrasted phenotypes between individuals reared in novel versus natal environments. Specifically, we contrasted phenotypic variation of fitness‐related traits between populations of *Physella* snails reared in natal versus multiple novel sites including sites with novel crayfish predators. Crayfish can induce phenotypic plasticity in snail morphology (Dewitt et al., 1999; Krist, 2002; Stevison et al., 2016), behavior (Alexander & Covich, 1991a, 1991b; Dewitt et al., 1999; Dickey & McCarthy, 2007), and life‐history traits (Crowl & Covich, 1990) in response to chemical cues released from crayfish feeding on conspecific snails. Because novel environments are predicted to increase phenotypic variability (Ghalambor et al., 2007; Lande, 2009, 2015; Paaby & Rockman, 2014; Rutherford, 2000; Schlichting, 2008), snails from populations lacking crayfish could express higher trait variance in habitats with crayfish than in their natal habitats. We contrasted variability between natal sites and sites with novel crayfish predators in specific growth rates (SGR) and two metrics of shell morphology that respond to crayfish presence and can defend against crayfish predation. Similarly, abiotic conditions in the novel environment could also induce greater variability in traits when conditions are sufficiently distinct from the natal environment. To determine whether novel habitats induced higher variability in population‐level expression of fitness‐related traits, we reciprocally transplanted six populations of juvenile *Physella* and contrasted variability in SGR and two metrics of shell morphology. Specifically, we addressed: (1) whether transplantation to sites with novel abiotic and biotic conditions increased variability in growth and morphological traits, (2) whether the presence of novel crayfish predators increased variability, and (3) which, if any, abiotic attributes led to the greatest change in trait variability.

## 2 MATERIALS AND METHODS

### 2.1 Establishing snail populations

In summer 2020, we surveyed all sites within a 96.6 km (60‐mile) radius of the University of Wyoming, Laramie where *Physella* occurred in a 2009 survey (Narr & Krist, 2020). The small radius reflected university‐imposed COVID‐19 restrictions on travel for field work. At the six sites where *Physella* occurred, we used minnow traps to assess crayfish occurrence and verified presence/absence of crayfish with surveys conducted by Wyoming Game and Fish in 2020. Crayfish (*Faxionus immunis*, *Faxonius neglectus*, and *Faxonius virilis*) occurred at three sites (Gelatt Lake, Crow Creek at Hereford Ranch, and Laramie River; Table 1) and were absent from the other three sites (Leazenby Lake inflow, beaver pond at Happy Jack Recreation Area, and Alsop Lake; Table 1). Snails from Happy Jack were identified as *Physella gyrina* and all other populations were identified as *Physella acuta* (identified to the species level by R.T. Dillon, Freshwater Gastropods of North America Project).

**TABLE 1:** Site attributes.

Site	Coordinates	Snail species	Crayfish status
Alsop Lake (AL)	41°23′36″N 105°47′21″W	*P. acuta*	Absent (−)
Crow Creek (CC)	41°07′38″N 104°41′57″W	*P. acuta*	Present (+)
Gelatt Lake (G)	41°14′14″N 105°50′06″W	*P. acuta*	Present (+)
Happy Jack Pond (HJ)	41°15′05″N 105°24′49″W	*P. gyrina*	Absent (−)
Laramie River (LR)	41°18′21″N 105°36′10″W	*P. acuta*	Present (+)
Leazenby Lake Inflow (LZ)	41°10′28″N 105°35′04″W	*P. acuta*	Absent (‐)

*Note*: Each snail population was reared in its natal site and in five, novel, transplant sites. Above, we list abbreviations for each population, their GPS coordinates, species of *Physella* present, and whether or not crayfish occurred. For this experiment, we used populations from all six sites for our comparisons of population‐level variability between natal and novel sites. However, to address the novel crayfish predator stimulus, we contrasted population‐level variability of Alsop (AL) and Leazenby (LZ) at their natal (crayfish absent) sites to three sites with novel crayfish predators (Crow Creek (CC), Gelatt (G), & Laramie River (LR)). Although crayfish were also absent at Happy Jack (HJ), we excluded this population from this analysis because a different species of *Physella* occurred there (*P. gyrina*) and previous studies suggest that snails are unlikely to respond to chemical cues from predation on heterospecifics (*P. acuta*; present at all other sites).

At each site, we collected approximately 20 adult snails, which we isolated in individual 237 mL plastic cups in the laboratory at the University of Wyoming. Twice a week, we fed all snails crushed TetraMin Tropical Flakes™ and changed water for each cup. For all snails that produced eggs, we reared offspring to obtain equal representation of each reproductive individual, and thus the genetic variation present in the samples that we collected for each population, to transplant at each site. To maintain low densities in cups, we moved offspring to new cups or aquaria depending on offspring numbers. Water changes in aquaria occurred twice daily using a timed water release and a flow through system. We used juvenile siblings, the offspring of a single snail, for the reciprocal transplant experiment. Studies of phenotypic plasticity typically use individual genotypes and these closely related genotypes were the closest proxy for genotypes in this sexually reproducing taxon. Thus siblings, whether selfed, full, or half (self‐fertilization or fertilization via sperm storage; Wethington & Dillon, 1991, 1997) allowed us to study the effects of novel environments on the phenotypic plasticity of closely related genotypes, but because we did not conduct genetic analyses, we do not know the amount of variance in relatedness within families. We referred to all siblings from a single mother as a family. To avoid confounding genetic diversity with transplant location, siblings from each family were equally represented at each transplanted site.

### 2.2 Experiment

In July and August 2021, we reared juvenile *P. acuta* and *P. gyrina* from each family and population at each site for 2 weeks. We individually marked snails from different families and populations with nail polish to track individual snails. At each site, we housed snails in 15 modified square plastic sandwich containers (289 cm^2^) with mesh windows on the tops and sides to permit water flow. Each container held six snails, and when sample sizes permitted, the snails were from six different populations. Due to population differences in survival and reproduction of field‐caught snails, the number of families in each population varied from two families (Alsop) to 10 families (Leazenby) and the number of individuals from each family varied from one to three at each novel site. No cage held multiple representatives of the same family. In contrast, because there is only one natal site relative to multiple novel sites, we included three representatives from each family (in different cages) at natal sites. For families with few siblings, we placed laboratory‐reared “filler” snails, which were not measured, to maintain the six‐snail density in each container. Therefore, the number of siblings varied by the number of reproductive individuals per population and the number of offspring produced per individual (family size). We placed 30 individuals in each site for the largest population, Leazenby, but for Alsop, we could only place four individuals at some sites. We characterized all non‐natal sites as novel to the transplanted snails because we assumed that environmental (biotic and abiotic) conditions are not identical between two disjunct sites; sites were not connected by water, were separated by a minimum of 17 km over land (maximum 96 km), and represented both lotic and lentic environments.

During the 14‐day duration of the experiment, we replenished food and removed dead snails and cleaned the mesh windows of algae, silt, and debris twice weekly. To feed the snails, we added approximately the same amount of food to each chamber at each site (epiphyton on macrophytes that occurred at each site). At three time intervals during the experiment at least 2 days apart, we measured temperature, conductivity, flow rate, and dissolved oxygen with a sonde (Yellow Springs Instrument, model 85) adjacent to the experimental containers. Midway through the experiment, we measured pH (Corning pH meter, Model 430), magnesium and calcium carbonate hardness (Hach Test Kit, Model 5‐EP), and ammonia (Hach Test Kit, Model NI‐8) from water samples adjacent to the experimental cages. To analyze food quality and quantity, we collected epiphyton samples at each site by scrubbing an equivalent surface area of submerged macrophytes from the same locations where we collected macrophytes to feed the snails. We collected samples of the slurry onto glass fiber filters (Pall Type A/E, 25 mm). We analyzed epiphyton phosphorus content using colorimetry and the ascorbic acid method (APHA, 1992) and carbon and nitrogen content at the University of Wyoming Stable Isotope Facility. Because of sample loss, we did not include chlorophyll *a* content of the epiphyton in our analyses.

Due to large distances between sites, we reared snails at paired sites at different times in summer 2021 (Gelatt Lake and Alsop Lake, June 28th–July 13th, Crow Creek and Leazenby Lake inflow July 15th–July 30th, and Happy Jack and Laramie River, August 2nd–August 17th). At the conclusion of each pair of trials, we measured and froze all snails.

Before and after each pair of trials, we measured three defense‐related traits that are commonly induced by crayfish and can protect snails from predation by crayfish (Crowl & Covich, 1990; Dewitt et al., 2000; Stevison et al., 2016): SGR, aperture shape, and aperture area. We estimated SGR by measuring shell length with an ocular micrometer (Leica S6E). To obtain consistent measurements, we used the same orientation and angle for all snails with the aperture facing upwards and the shell slightly rotated to measure maximum shell length. To obtain biomass, we developed mass‐length regressions for each population following Benke et al. (1999). We measured aperture width, length, and area with the aperture facing upward and parallel with the measuring surface. We used an image‐processing microscope (Olympus SZX2‐ILLT) and ImageJ software (Schneider et al., 2012) to analyze shell morphology for each snail. To account for variation in snail size, we calculated aperture shape as the ratio of aperture width to aperture length and aperture area as the ratio of aperture area to shell length.

We also measured a proxy of shell thickness (crush force). However, we subsequently omitted these data from the analyses because: (1) crayfish are primarily aperture entry predators, thus crush force is not directly relevant to defense from crayfish (Alexander & Covich, 1991a, 1991b; Dewitt et al., 2000), and (2) in a review, Bourdeau et al. (2015) demonstrated that freshwater snails preferentially alter shell dimensions over increasing shell thickness because calcium is more often limited in freshwater than in marine environments.

### 2.3 Statistical analysis

To contrast population‐level expression of trait variability between natal and novel sites, we re‐sampled our data to obtain the likelihood of our observed ratios of variance compared with permuted ratios. We calculated observed ratios as the variance observed in the novel environment divided by the variance observed in the natal environment (var(novel)/var(natal)). We calculated permuted ratios by randomly re‐shuffling, without replacement, the combined data into either novel or natal categories 1000 times, controlling for the total sample size of observed distributions for each population. This randomization method created a null distribution of values which we compared to the observed ratios of natal to novel variation. Then, we compared the observed variance ratio to the distribution of permuted variance ratios and reported the *p*‐value as the sum of the simulated variance ratios that were greater than the observed variance ratio, divided by the sample size. High variance ratios indicated greater variance than expected by chance in novel habitats than natal habitats based on the distribution of observed values relative to permuted values. We performed all statistical analyses in R version 2021.09.0+351 (R Core Team, 2022).

We used permutation tests because parametric statistics were not appropriate for our data or for the focus of our research questions. In our experiment, sample sizes varied among families and populations, were sometimes small, and were non‐normally distributed for many populations. Also, we had unequal variances between natal and novel sites, which we did not want to amend with data transformations because we predicted differences in variance between fitness‐related traits in natal and novel sites. Permuting our observations allowed us to leverage all our data, rather than reducing our observations to a few measures of variability (e.g., variance or coefficient of variation). Our method also allowed us to account for differences among populations in sample size. Finally, because of small sample sizes in some populations we could not build hierarchical models that included both family and population. To address this limitation, we represented families equally among all transplant sites.

To address whether transplantation to any novel site increased variability in growth and morphological traits (Objective 1), we contrasted the variability of all populations in their natal sites to the variability of all populations in novel sites. To address whether the presence of novel crayfish predators increased variability in defense‐related traits (Objective 2), we contrasted population‐level variability in crayfish‐absent populations of *P. acuta* between their natal sites and sites with novel crayfish. We omitted one site from this analysis (Happy Jack) because the snails were a different species, *P. gyrina*, and thus less likely to respond to predation of the heterospecific, *P. acuta*, which occurred at every other site (Dalesman et al., 2007).

To determine which abiotic attributes of sites induced the highest trait variability (Objective 3), we used permutation tests to contrast variability between the natal and most distinct site for each attribute. We did not combine abiotic variables using ordination methods because we sought to understand which specific abiotic attributes drove high variability (Objective 3). Because the most distinct abiotic attributes should expose the greatest trait variability (Ghalambor et al., 2007; Lande, 2009), we predicted that the greatest differences in abiotic attributes between natal and novel sites would be associated with the greatest trait variation (darkest values in Figure 4). To assess the accuracy of our predictions for each abiotic attribute (e.g., pH), we tallied all instances where greater trait variation corresponded with the most distinct abiotic attribute and divided that count by the number of total predicted cases. We used permutation tests (as described above) to determine whether high variability in novel sites was associated with distinct (most different from natal) abiotic attributes, for each response variable.

## 3 RESULTS

### 3.1 Do novel environments increase variability in growth and morphology? (Objective 1)

**FIGURE 1:**
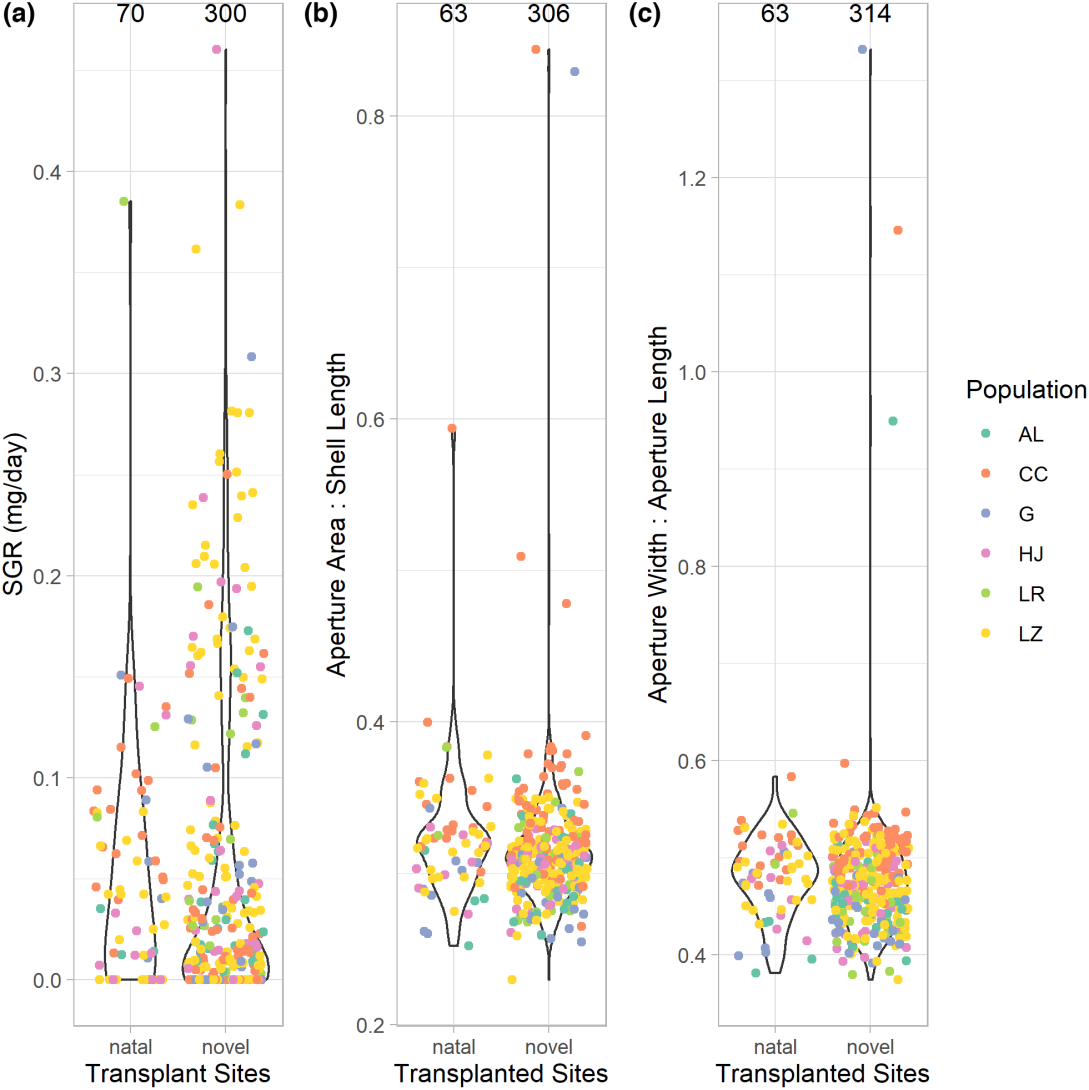
Comparison of trait variability in snails in natal and all transplanted novel sites. Snails expressed much greater variability in SGRs in novel, transplanted sites relative to their natal site (a; *p* < .001). Populations expressed moderately higher variability in aperture area in novel sites relative to their natal site (b; *p* ≤ .020), and no difference in variability in aperture shape (ratio of aperture width to aperture length) between natal and novel sites (c; *p* > .5). Total sample sizes are shown above each violin plot for natal and novel transplants. Each dot represents one individual, and different colored dots represent different populations (AL = Alsop, CC = Crow Creek, G = Gelatt, HJ = Happy Jack, LR = Laramie River, and LZ = Leazenby).

For snails from all populations combined (crayfish absent and crayfish present), both SGR (*p* < .001; Figure 1a) and aperture area (*p* < .020; Figure 1b) were more variable in the novel than natal environments. Aperture shape did not differ between natal and novel environments (*p* > .5; Figure 1c).

**FIGURE 2:**
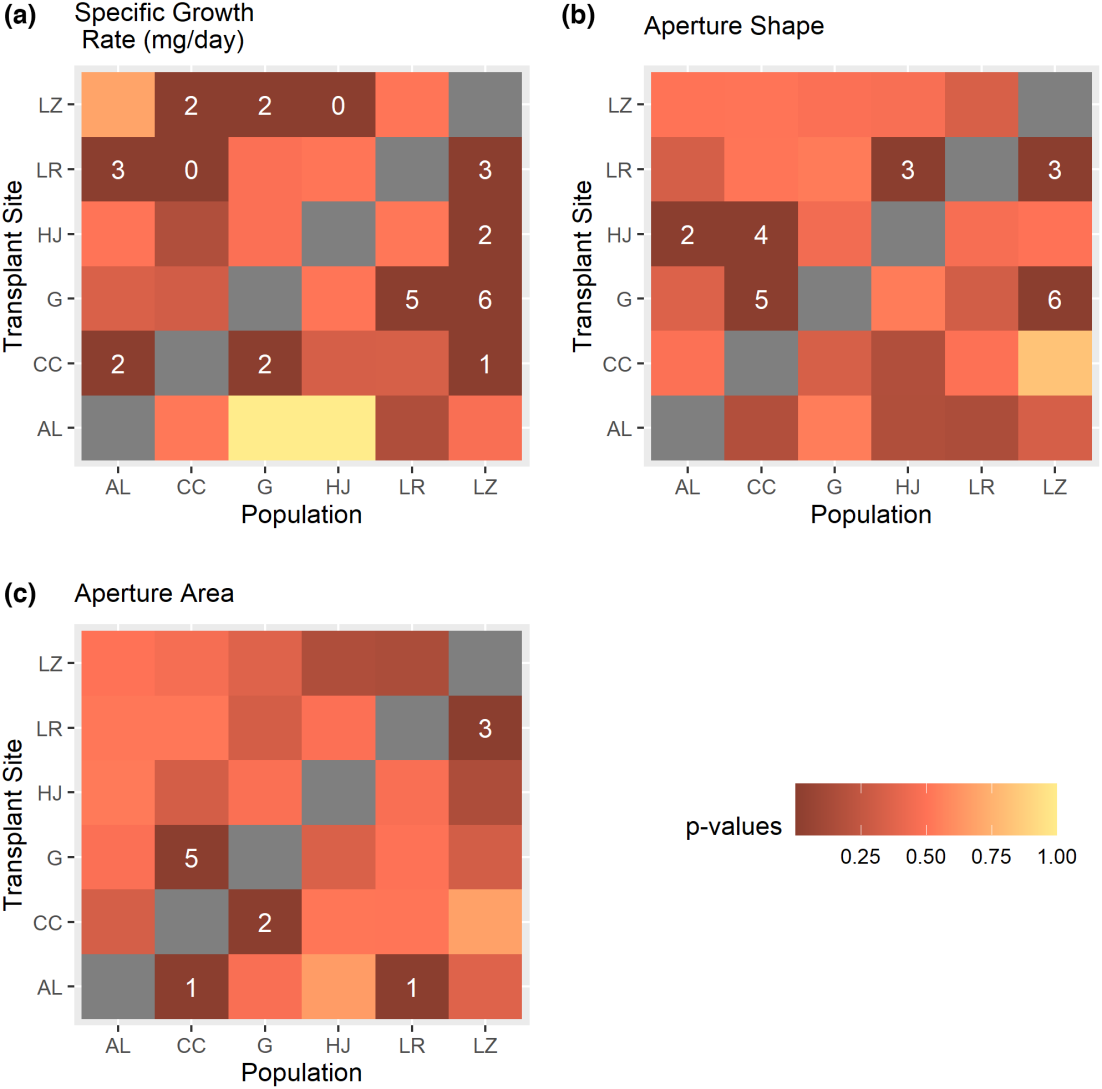
Observed variability and number of distinct abiotic attributes in novel sites. In 91% of cases where variability in novel sites exceeded natal sites, the associated novel site possessed one or more abiotic attributes that exhibited the greatest difference from the natal site. The numbers in tiles indicate how many abiotic attributes in the novel site differed maximally from the natal site in cases where variability in novel sites exceeded natal sites (*p* < .05; darkest tiles). Populations and sites are abbreviated (AL = Alsop, CC = Crow Creek, G = Gelatt, HJ = Happy Jack, LR = Laramie River, and LZ = Leazenby).

Collectively, novel abiotic attributes had widespread effects on variability in snail traits. In 91% of cases (21/23) where variability in the novel environment exceeded variation in the natal environment, at least one abiotic attribute exhibited the greatest difference from the natal site (Figure 2).

### 3.2 Does the presence of novel crayfish predators increase variability in growth and morphology? (Objective 2)

**FIGURE 3:**
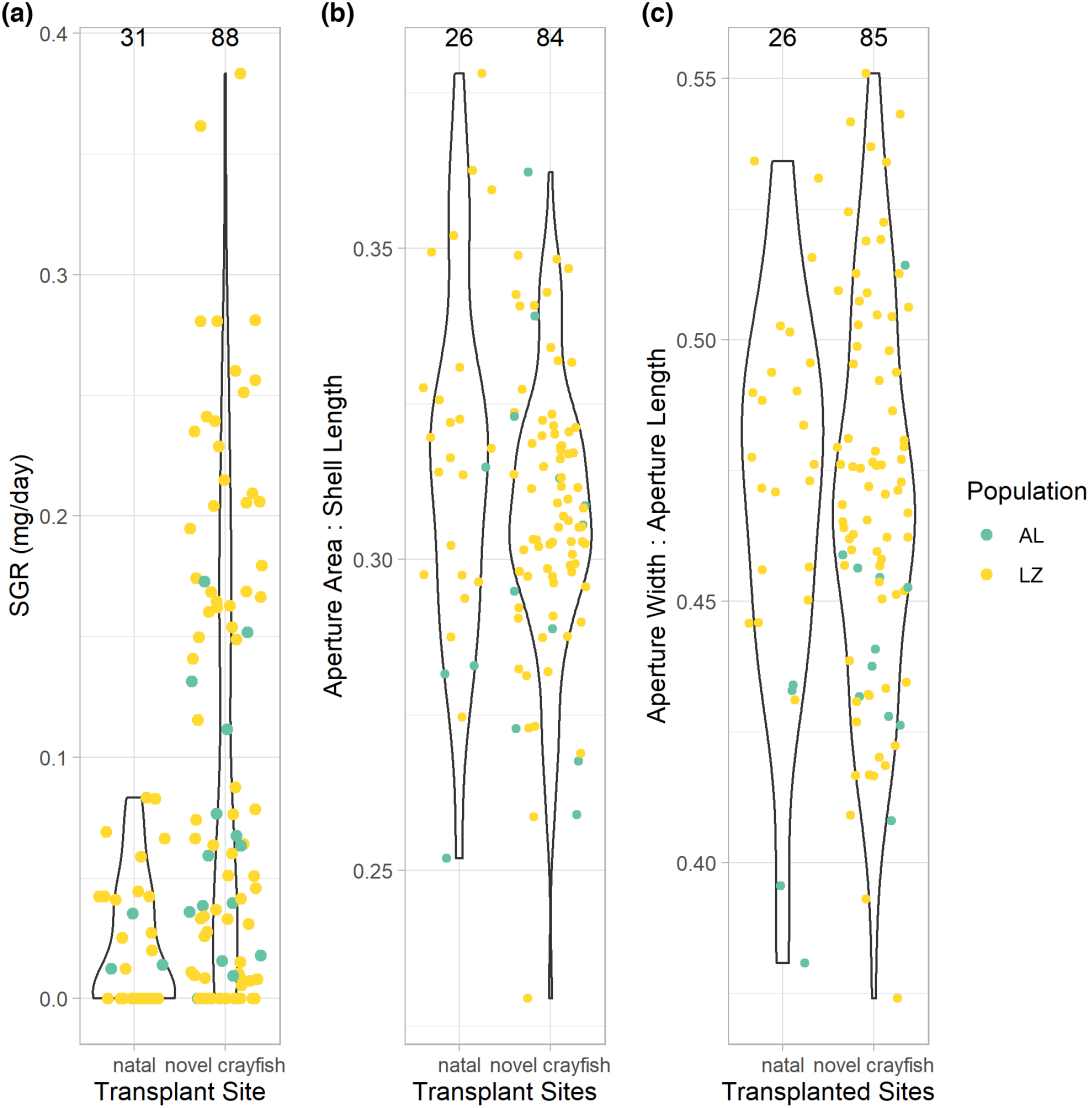
Comparison of trait variability in snails between natal and transplanted sites with novel crayfish predators. Snails from two sites lacking crayfish (Alsop Lake, AL; Leazenby Lake Inflow, LZ) expressed greater variability in SGRs (a; *p* ≤ .015) in sites with novel crayfish predators (Crow Creek, Gelatt Lake, and Laramie River) relative to their natal sites. Neither aperture shape (b; ratio of aperture width to aperture length) nor aperture area (c; ratio of aperture area over shell length) exhibited higher variability in novel (*p* > .30 and *p* > .10, respectively) relative to natal sites. Total sample sizes are shown above each violin plot.

Populations of snails from sites lacking crayfish expressed much higher variability in SGR in novel sites than in their natal sites (*p* ≤ .015; Figure 3a). Variability did not differ between natal and novel sites for aperture area (*p* > .10; Figure 3b) or aperture shape (*p* > .30; Figure 3c).

### 3.3 Which abiotic attributes lead to the greatest change in trait variability? (Objective 3)

**FIGURE 4:**
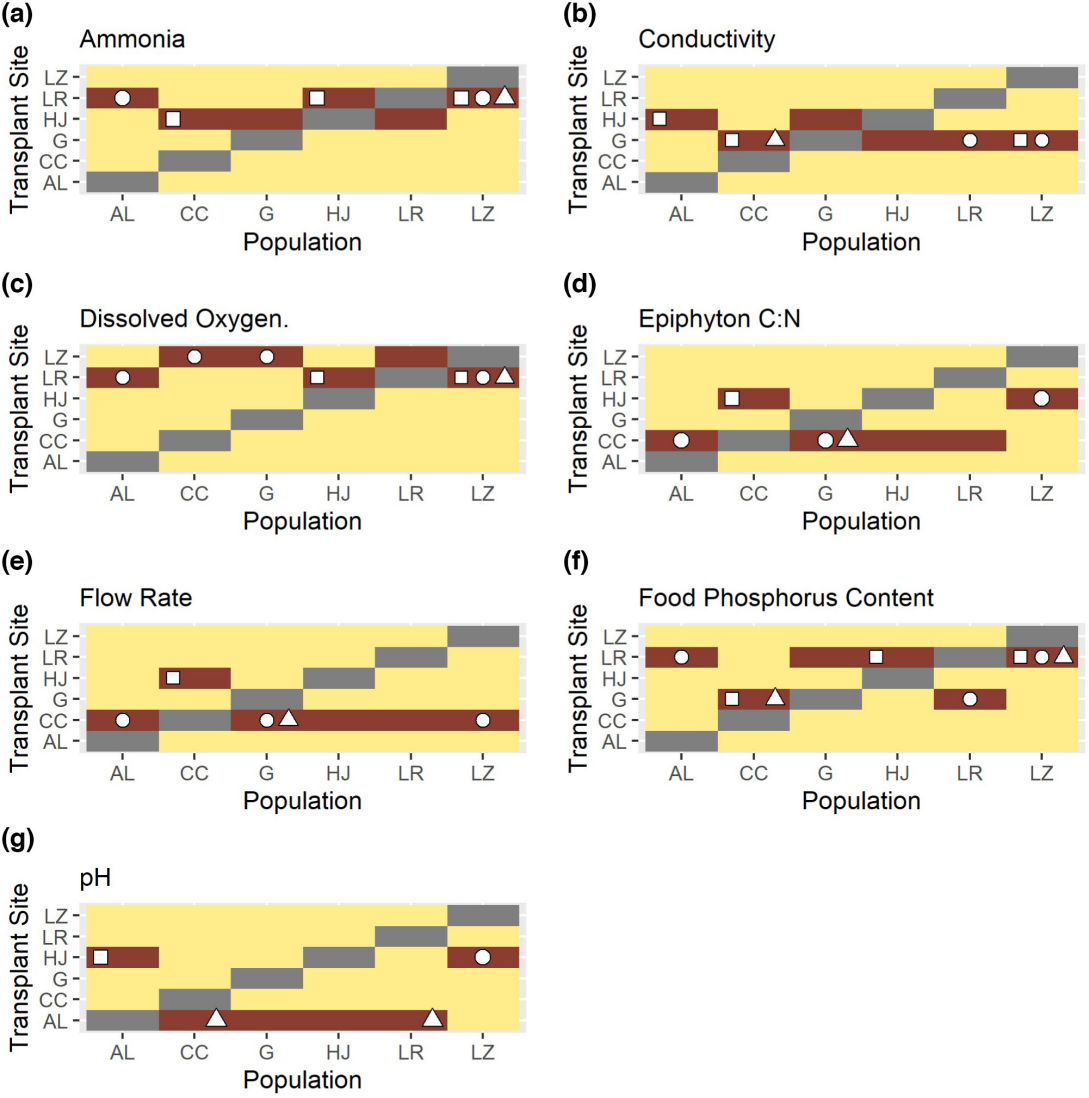
Most associated abiotic attributes. For each combination of population and transplant site, we colored sites that were the most distinct for each population in dark red to highlight novel sites that were predicted to induce the greatest trait variability. Shapes (circle = SGR, square = aperture shape, triangle = aperture area) indicate where predictions aligned with our observations of greater trait variability in the novel compared with the natal site. These six abiotic attributes were associated with ≥ 67% of the observed high variation in at least one fitness‐related trait. Populations and transplant sites are abbreviated (AL = Alsop, CC = Crow Creek, G = Gelatt, HJ = Happy Jack, LR = Laramie River, and LZ = Leazenby).

For growth and morphological traits combined, observed high novel variation in one or more traits corresponded with distinct levels of dissolved oxygen and phosphorus content of food (epiphyton) in 83% of predicted cases (Figure 4) and with ammonia, conductivity, epiphyton C:N, flow rate, and pH in 67% of predicted cases (Figure 4).

**FIGURE 5:**
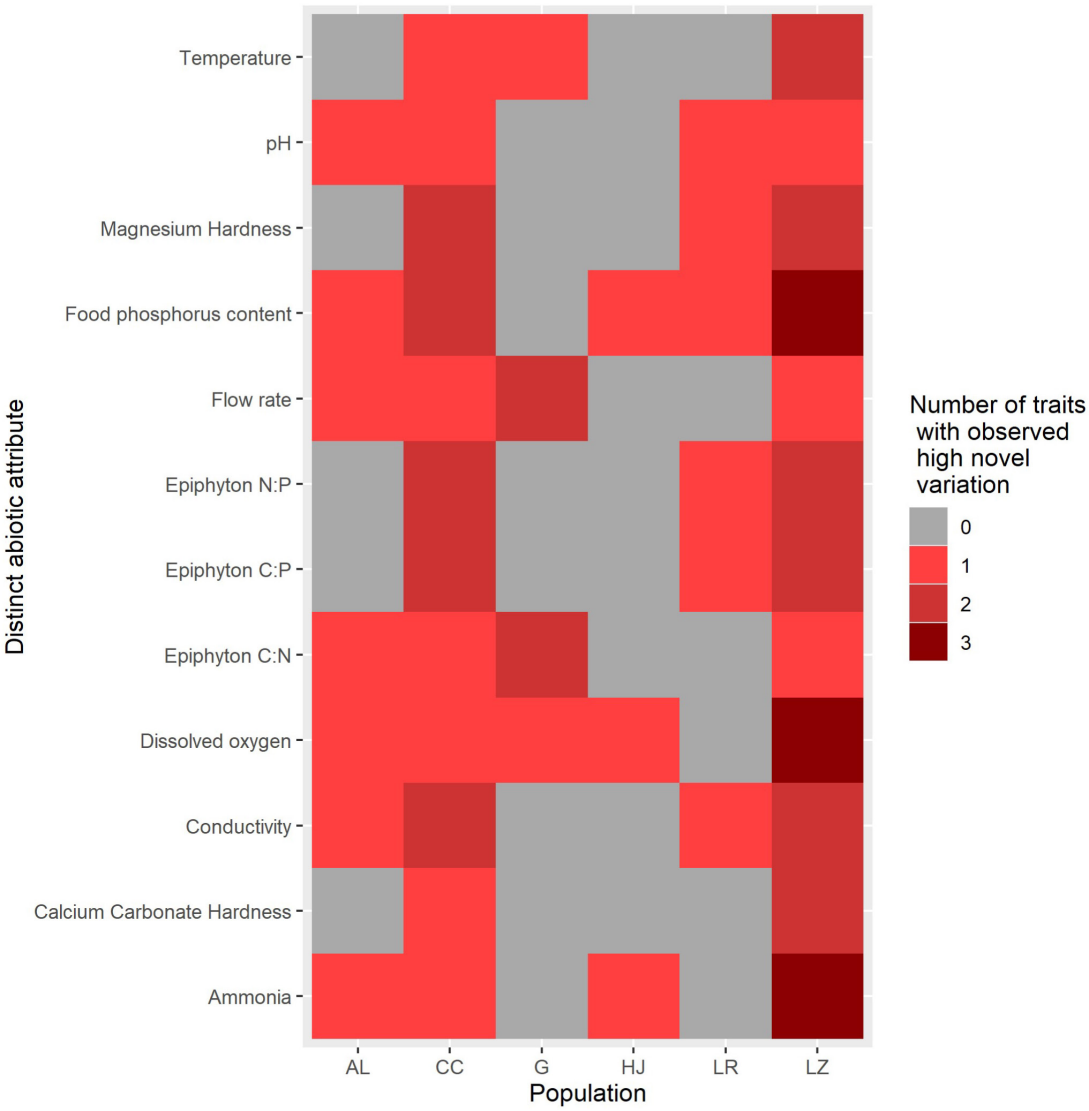
Population differences in responsivity to distinct abiotic attributes. On the red scale, darker colors indicate more fitness‐related traits exhibited greater variation in novel sites for each distinct abiotic attribute. Leazenby (LZ) and Crow Creek (CC) populations consistently exhibited high trait variability in novel sites with at least one trait responding with high trait variability to every distinct abiotic attribute. In contrast, Happy Jack (HJ) and Gelatt (G) responded less to distinct abiotic attributes. Alsop (AL) and Laramie River (LR) populations were moderately responsive to abiotic attributes. No distinct abiotic attribute induced high novel variability in all populations; however, food phosphorus content and dissolved oxygen induced greater novel variability in five out of six populations.

Variability in SGR was the greatest when dissolved oxygen, epiphyton C:N, flow rate, food phosphorus content, and temperature were the most distinct from the natal environment (>50% correspondence between predicted and observed instances of high variation; Figures 4 and 5). For variability in aperture shape, distinct ammonia, conductivity, and food phosphorus content most often corresponded (>50%).

## 4 DISCUSSION

Consistent with theory, novel conditions increased variation in fitness‐related traits of *Physella*. The presence of novel crayfish predators increased variation in *P. acuta* growth rates and novel abiotic conditions increased variation in *P. acuta* and *P. gyrina* growth rates, and aperture area. Dissolved oxygen and food phosphorus content were most consistently associated with high variation in novel sites. High variation in fitness‐related traits at the population level may allow *Physella* populations, and other organisms, to persist despite large and rapid changes in the environment.

Our results are consistent with many previous studies demonstrating that *Physella* can exhibit phenotypic plasticity in response to novel stimuli. *Physella* can respond almost immediately to predator cues with behavioral plasticity (Alexander & Covich, 1991a, 1991b; Dewitt et al., 1999; Dickey & McCarthy, 2007; Turner et al., 2000), and longer term, *Physella* and other freshwater (Brönmark et al., 2011; Crowl & Covich, 1990; Dewitt et al., 2000; Krist, 2002), and marine (Trussell, 2000) snails can respond with life history and morphological plasticity. For example, *Physella* exposed to crayfish predator cues can quickly display predator avoidance behaviors (behavioral plasticity) by crawling out of water for multiple hours (Alexander & Covich, 1991a, 1991b). Long‐term exposure to crayfish cues can alter *Physella* growth rates and delay reproduction (Crowl & Covich, 1990). Additionally, predators can induce defensive shell shapes in multiple freshwater snail species (*Radix balthica*: Brönmark et al., 2011; *Elimia livescens*: Krist, 2002; *Planorbella trivolvis*: Hoverman & Relyea, 2009) within the lifetime of an individual snail.

Increased variation in novel sites likely resulted from expression of genotypic variants previously unexpressed in the natal site (Buckley et al., 2010; Waddington, 1942). Buffering mechanisms present in natal sites dissolve when released from natal regulatory cues or in response to stressful, novel conditions (Paaby & Rockman, 2014; Rutherford, 2000; Schlichting, 2008). Release from buffering mechanisms can increase variance in traits leading to negative, neutral, or positive effects on individual fitness in the new environment. Effects can be beneficial for populations by increasing the range of values for fitness‐related traits, thereby increasing the probability that one or more variant will be adaptive and allowing selection to act on a greater array of phenotypes (Ghalambor et al., 2007; Rutherford, 2000; Rutherford & Henikoff, 2003).

Our results are consistent with a number of studies demonstrating higher variability in populations exposed to novel conditions. Temperature shocks induced increased variation in wing spot patterns in the pale grass blue butterfly (*Zizeeria maha*; Buckley et al., 2010) and increased morphological variation in female sperm storage organs in the yellow dung fly (*Scathophage stercoraria*; Berger et al., 2011). Male brown trout (*Salmo trutta*) exposed to stressful water pH displayed increased variability in sperm performance (Purchase & Moreau, 2012). In contrast to most studies of phenotypic variation in novel or stressful environments conducted in laboratory settings and focused on a single manipulated attribute, our study examined trait variability in response to an array of novel conditions in field experiments (reciprocal transplants).

### 4.1 Do novel environments increase variability in growth and morphology?

Contrasting population‐level variability of all traits between all natal and novel sites, we found that transplantation to novel environments very strongly increased variability in growth rates (sensu Muff et al., 2022), moderately increased variability in aperture area (*p* ≤ .02) but had no effect on aperture shape (Figure 1). Induced variation may help populations survive novel environments and rapid changes to their natal habitats (Ghalambor et al., 2007). Unless all individuals express plasticity that is maladaptive, precluding any individuals from expressing trait values that rescue the population in novel environments, greater variation increases the probability that some individuals in a population will possess adaptive traits that permit the population to persist in the new environment (Ghalambor et al., 2007; Rutherford, 2000; Rutherford & Henikoff, 2003). In our study, we refer to population‐level plasticity as intrapopulation variation, because the latter is the composite of phenotypic plasticity of all individuals from a population. Our results also suggest that at least some attributes of novel sites differed sufficiently from natal environments to elicit increased trait variability among individuals in populations.

### 4.2 Does the presence of novel crayfish predators increase variability in growth and morphology?

*Physella acuta* from populations lacking crayfish predators, exhibited much higher variability in growth rates in sites where crayfish occurred than in their natal sites (Figure 3). Thus, we found strong evidence (*p* ≤ .015) that crayfish predators induced greater variability in growth rate in snail populations without a history of predation by crayfish. Intrapopulation variation in SGR could facilitate population persistence of *Physella* in the presence of novel crayfish predators by allowing some individuals to increase growth rates quickly to attain a size large enough to deter predation (Covich, 2010; Crowl & Covich, 1990).

Because sites with crayfish also differed, at least somewhat, in all abiotic characteristics relative to the natal environment, any differences between sites, whether biotic or abiotic, could have increased variability in snail growth rates. For example, snails from Leazenby Lake inflow transplanted to each of the sites where crayfish occurred also experienced the greatest difference in at least one abiotic characteristic per site relative to their natal site: Crow Creek differed most in flow rate, Gelatt Lake in conductivity, calcium carbonate concentration, and magnesium hardness, and the Laramie River had the most distinct levels of dissolved oxygen, ammonia, and food phosphorus content. Thus, these distinct abiotic attributes could have contributed to, or superseded, the effect of crayfish presence for the Leazenby population in these novel transplant sites.

In contrast to growth rates, we found limited evidence that variability in shell morphology was greater at sites with novel crayfish predators. However, snails from Leazenby exhibited higher variation in novel sites for all three traits (SGR, aperture shape, and aperture area; *p* < .001). Because sample sizes were much larger for Leazenby than Alsop, perhaps we did not find altered variability in shell shape for the combined analysis or for Alsop alone because of reduced statistical power. Also, we may have found a stronger response in shell morphology if our experiment had been longer; relative to SGR, alterations in shell morphology require more growth, and hence more time, to achieve. Previous experiments showing changes in snail shell morphology were 4–20 weeks in duration (Bukowski & Auld, 2014; Krist, 2002; Zalizniak et al., 2009), 2–18 weeks longer in duration than our experiment. Additionally, for morphological traits there can be significant lag times between the occurrence of an environmental cue and the organismal response through phenotypic plasticity (DeWitt et al., 1998).

### 4.3 Which abiotic attributes lead to the greatest change in trait variability?

When snails exhibited higher variability in the novel relative to the natal environment, in nearly every case (21/23 cases) at least one abiotic attribute at the novel site was the most distinct from natal conditions (Figure 2). Thus, whether increased variability was driven by the most distinct abiotic characteristic or some combination of measured or unmeasured abiotic or biotic characteristics, populations responded to some attribute of the novel transplanted sites. Abiotic attributes closely tied to fitness can serve as important environmental cues for plastic responses (Bourdeau et al., 2015).

In over 50% of cases where we observed variation in SGR and aperture shape was greater in the novel site than the natal site, the greatest difference between the two sites was in food phosphorus content (Figure 4). Dietary phosphorus affects snail fitness because it is required for growth (Osenberg, 1989; Sterner & Elser, 2002), is often limiting for primary consumers in freshwater (Cross et al., 2005) and can reduce fitness of snails at high levels (Elser et al., 2006).

Distinct levels of dissolved oxygen, temperature, flow rate, and epiphyton C:N were also associated with variation in SGR in ≥50% of cases where observed variation of SGR in novel sites exceeded natal sites (Figure 4). Dissolved oxygen can contribute to distributions of pulmonate snails (Ofoezie, 1999) and may also be correlated with other, unmeasured attributes of the novel environment. High temperatures increase growth rates in *P. virgata* (McMahon, 1975) and *P. acuta* can outgrow *P. frontalis* in higher temperatures (Früh et al., 2017). Dislodging rates of freshwater snails increase with snail size (*Physella propinqua*; Moore, 1964), suggesting that low growth rates may be beneficial in sites with high flow rates. Even though we did not find an effect of flow rate on variation in shell morphology, flow rate can also induce changes in *Physella* shell shapes; for example, aperture width and foot size increased in high velocity waters (Gustafson et al., 2014). Epiphytic C:N content can also be important to growth and reproduction. Decreased protein to cellulose content of food caused *P. gyrina* to shift allocation from growth to reproduction (Rollo & Hawryluk, 1988), suggesting that dietary C:N can alter allocation to life‐history traits in *Physella*.

Because pH is often correlated with freshwater mollusk abundance (Cieplok & Spyra, 2020; Lewin et al., 2015; Spyra, 2010), our finding that pH was associated with either high variability in SGR, or aperture shape, or aperture area (one trait per population) is consistent with previous studies showing the importance of pH to mollusks. Distinct values of conductivity and ammonia were most often associated specifically with high observed variation in aperture shape. Conductivity commonly affects snail fitness and distribution (Larson et al., 2020; Lewin et al., 2015) and conductivity is correlated with shell shape in the freshwater snail, *Elimia livescens* (Cazenave & Zanatta, 2016). Although multiple studies have demonstrated the effect of ammonia on snail growth rates, very few studies have analyzed how ammonia affects morphology. Although high ammonia concentrations are correlated with oxidative stress in *Physa acuta* (an invalid synonym for *Physella acuta*; Integrated Taxonomic Information System, www.itis.gov; Sedeño‐Díaz & López‐López, 2022), all transplant sites in our study possessed relatively low levels of ammonia (no more than 0.17 mg ammonia/L; lower than all ammonia concentrations in Sedeño‐Díaz & López‐López, 2022). Additionally, at concentrations similar to our transplant sites, no effects of ammonia concentrations occurred on *Physella* growth or survival (Besser et al., 2016). However, in every case where high variation corresponded with the most distinct ammonia levels, other abiotic attributes (epiphyton C:N content, flow rate, food phosphorus content, and dissolved oxygen) covaried with ammonia suggesting that some other abiotic attribute could have induced greater variability.

### 4.4 Differences among populations

Some populations exhibited greater plasticity than others in response to specific novel conditions. For example, the Leazenby population exhibited greater variability in one or more traits corresponding to all 12 distinct abiotic attributes (*p* < .05; Figure 5), yet snails from the Laramie River exhibited greater variability in only one trait in response to six distinct abiotic variables (*p* < .05; Figure 5). Differences among population responsiveness to novel conditions may be explained by the variability of abiotic conditions in natal sites. Leazenby Lake inflow is very stable in temperature and hydrology because the water source is an environmental laboratory. Thus, perhaps the greater variability exhibited in snails from Leazenby to novel conditions occurred because their natal conditions are relatively invariant, making even small deviations from the natal range sufficient to be perceived as distinct novel conditions to snails from this population. In contrast, the Laramie River site is an irrigation canal which we observed to experience a greater range of water levels and flow rates (and likely temperatures). Thus, perhaps snails from Laramie River require larger deviations to induce variability given the greater range of typical natal conditions that they experience.

Additionally, no abiotic attribute induced increased plasticity in all populations, and certain abiotic attributes induced variation in a select few populations. For example, shell morphology variation increased for the Crow Creek population in sites with the most distinct conductivity, magnesium hardness, and food phosphorus content, whereas the Gelatt population did not increase variation in response to any of these distinct abiotic attributes (Figure 5). Different populations may vary in sensitivity to environmental cues and different degrees of phenotypic plasticity due to differences in evolutionary history (Bourdeau et al., 2015; DeWitt et al., 1998). Additionally, for some populations, abiotic attributes may not have been distinct enough from natal conditions to elicit greater variability.

### 4.5 Limitations

Small sample sizes and few study populations limited the statistical power of our analyses and thus the strength of inference of our experiment. We were limited to six study populations by university‐imposed COVID‐19 restrictions on the geographic area where we could work (60 mile radius of Laramie, WY). Also, high mortality of parental snails reduced the number of families per population and low‐offspring production of many surviving parental snails limited the sample sizes of snails from each family. As a result, some families had few replicates in natal and across novel sites. However, each family was represented across all transplant sites (equal genetic variation across sites). Additionally, for our comparisons of variation in natal sites against novel sites, we had lower sample sizes for natal sites than sample sizes from all novel transplant sites combined. Because variation is expected to be higher in smaller samples (Well et al., 1990), our study design was biased to find higher variation in natal sites than in novel sites. Yet, despite these limitations, we found greater variation in two‐thirds of our fitness‐related traits at novel sites.

We found strong evidence that freshwater snails increase intrapopulation variability in traits related to fitness in response to a diverse array of environmental conditions, suggesting that plasticity could be a powerful tool for population persistence in the face of rapid environmental change. However, plasticity, alone, may not be sufficient to buffer thermal tolerance of ectotherms from climate change (Gunderson et al., 2017; Gunderson & Stillman, 2015) but may contribute to or be sufficient to buffer populations from other environmental changes.

### 4.6 Importance

Warming waters, introduced species, changes in flow regimes, and pollution threaten freshwater ecosystems. Climate change affects a wide range of organisms and ecosystems. Rapidly changing environmental conditions can cause losses in biodiversity and changes in species distributions in any ecosystem (Alahuhta et al., 2019; Parmesan et al., 2003; Woodward et al., 2010). To persist, species must be able to respond to novel conditions.

Freshwater snails are vulnerable to many anthropogenic disturbances. Increasing water temperatures, changes in precipitation, and a decrease in the global water supply change native habitats and reduce the ranges of suitable habitat for many native snail species (Cordellier et al., 2012). Additionally, climate change facilitates the invasion of competitor species and predators into disturbed habitats with native snail species (Johnson et al., 2009; Rahel et al., 2008). Non‐native crayfish have been introduced in every major biogeographical region and affect aquatic ecosystems and their snail prey in introduced ranges (Lodge et al., 2012). In North America, the invasive rusty crayfish, *Faxonius rusticus* (formerly *Orconectes rusticus*), has profound impacts on the lake and river ecosystems (Johnson et al., 2009; Kuhlmann, 2021). In invaded habitats, *F. rusticus* drastically reduces the abundance of native snails via predation (Johnson et al., 2009; Wilson et al., 2004). If native snails can respond with increased variation in defense‐related traits, they may be able to persist in their natal environments when novel predators invade, or other novel conditions occur.

We show that freshwater snails can react to novel conditions by increasing phenotypic variation of fitness‐related traits. Variation may promote population persistence by amplifying the diversity of phenotypes. Although some phenotypes will be maladaptive in the new environment, enhanced variability also increases the probability that some novel phenotypes will survive and reproduce in the new environment. Thus, the release of such variation can instigate the evolution of the appropriate genetic architecture required for a population to move to an optimum phenotype (Schlichting, 2008). In turn, increased variability can allow a population to shift towards the optimum phenotype in the new environment (Ghalambor et al., 2007), providing natural selection with a large array of phenotypes on which to act. The extent to which other taxa in natural conditions can harness increased variability to cope with novel, often rapid anthropogenic environmental change is unknown, but laboratory studies suggest that this phenomenon is common and phylogenetically widespread (Berger et al., 2011; Buckley et al., 2010; Purchase & Moreau, 2012). In this case, variability could provide an invaluable tool for organisms to survive and persist in our rapidly changing, human‐altered world.

## AUTHOR CONTRIBUTIONS

**Arielle W. Balph:** Conceptualization (supporting); data curation (lead); formal analysis (lead); funding acquisition (lead); investigation (lead); methodology (supporting); project administration (lead); resources (supporting); software (supporting); supervision (supporting); validation (supporting); visualization (lead); writing – original draft (lead); writing – review and editing (supporting). **Amy C. Krist:** Conceptualization (lead); formal analysis (equal); funding acquisition (equal); methodology (equal); resources (lead); supervision (lead); validation (lead); writing – review and editing (equal).

## FUNDING INFORMATION

This experiment was primarily funded by the University of Wyoming‐National Park Service small grants program and some lab analyses were funded by the National Science Foundation EPSCoR program (OIA‐2019596).

## CONFLICT OF INTEREST STATEMENT

The authors declare they have no conflict of interest.

## Data Availability

Data are located on Dryad: doi: 10.5061/dryad.x3ffbg7qh.
